# Sir Henry Dale (1875–1968)

**DOI:** 10.1007/s00415-024-12309-0

**Published:** 2024-03-27

**Authors:** Stefano Sandrone

**Affiliations:** Department of Brain Sciences, Imperial College London, London, UK

**Fig. 1:**
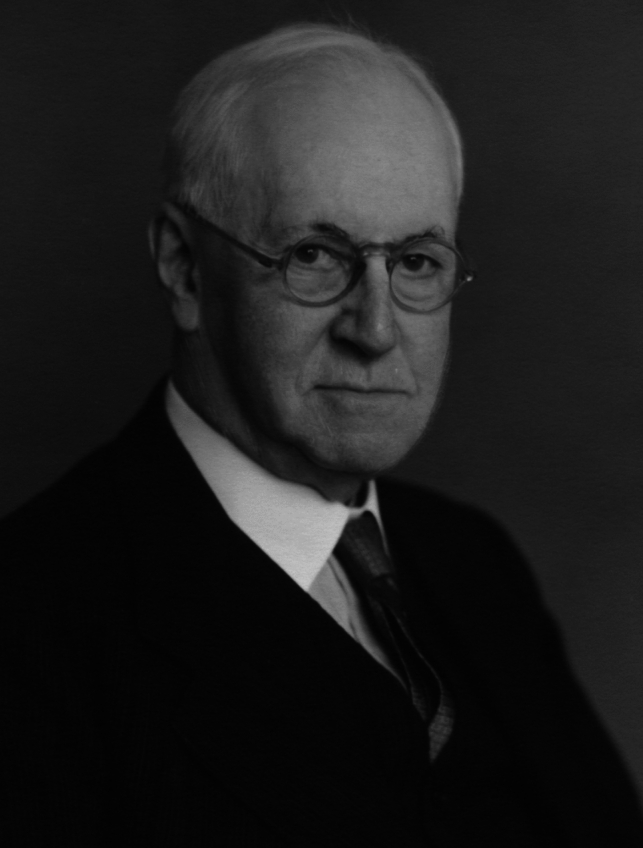
Portrait photo of Sir Henry Hallett Dale by Walter Stoneman, 1953. Credit: Photographs Collection, National Portrait Gallery, London. Reproduced by permission

Sir Henry Dale (Fig. 1) was ‘one of the most brilliant medical scientists of all time, whose discoveries in physiology and pharmacology laid foundations for vast advances in clinical medicine’ [1]. He was born in London on 9 June 1875 as the third child of seven. Dale’s mother, Frances Ann Hallett, came from a Devonshire family and met Charles James Dale, a businessman, at a Methodist Chapel [2]. Since 1883, Dale attended Tollington Park College, where Edward Albert Butler, a zoologist and vice principal, emphasised science teaching [2].

At the 1891 Annual Wesleyan Methodist Conference, Dale’s father introduced Henry to W. F. Moulton, headmaster of the Leys School in Cambridge, a new Methodist school with a focus on science and a physical alongside intellectual proximity to university laboratories, which hosted former pupils working at the University [2]. Dale was invited to take the Leys exams, passed them, and secured a scholarship [2]. At Leys (1891–1894), he was exposed to the latest physiology advancements by Alfred Hutchinson, who was finalising Foster’s new textbook. Dale next won a scholarship to attend Trinity College.

He graduated from the Natural Sciences Tripos with a specialisation in physiology and zoology and was a Coutts-Trotter physiology student under J. N. Langley [3]. Dale’s clarity of ideas [4] and language skills meant that many of his manuscripts in the *Journal of Physiology* were a delight to its readership and rarely, if ever, influenced by the ‘robust’ style of Langley, the Editor-in-Chief—although Dale’s readiness to edit papers authored by peers ‘was not always appreciated’ [2].

In 1900, St. Bartholomew’s Hospital in London was the place for his clinical medical segment; Dale obtained a B.Chir. in 1903 and an M.D. from Cambridge in 1909 [3]. Thanks to the George Henry Lewes Studentship, Dale joined University College London (UCL). In Ernest Starling’s laboratory, he met Otto Loewi, who was about to accept an academic position in Austria. Dale briefly worked with Paul Ehrlich in Frankfurt am Main and became a Sharpey Scholar at UCL for just 6 months, as, in 1904, Dale withdrew from the programme to work for the pharmaceutical manufacturer Henry Wellcome at Wellcome Physiological Research Laboratories [5, 6].

The year 1904 was crucial personally and professionally: Thomas Elliott, a Cambridge-based fellow scientist, suggested that adrenaline might be the chemical released after sympathetic nerve stimulation [5, 7, 8]; his ideas were met with scepticism. Dale started working on the ergot of rye (used to hasten slow labour in obstetric practise), accepting a commercially driven suggestion by Wellcome [6], but was free to pursue his scientific interests. That year, Dale married Ellen Harriett Hallett, and the couple parented two daughters and a son. Two years later, Dale became director of the Laboratories. He completed a wide range of investigations, from the discovery of ergotoxine to demonstrating that preparations of ergot contained tyramine and histamine, from elucidating the action of histamine on capillary to showing that the posterior pituitary extract has an oxytocic effect.

However, it was in 1913 that, following a ‘lucky accident’, Dale isolated (with Arthur Ewins) naturally occurring acetylcholine, which was a ‘synthetic curiosity’ at that time [8] as ‘rare contaminant in a batch of ergot’, with little direct support for any physiological significance [6]. With Harold Dudley, somewhat unexpectedly, they found acetylcholine as a natural constituent of the mammalian body while looking for endogenous histamine [6]. In 1921, Loewi provided evidence of the chemical nature of synaptic transmission, and the so-called *Vagusstoff* was identified as acetylcholine [8]. This was a paradigm shift, as the dominant theory spearheaded by John Eccles assumed that neural transmission was an electrical phenomenon, not a chemical one [5]. Dale used the words ‘cholinergic’ and ‘adrenergic’ to designate nerve fibres by the nature of the chemical, going beyond the anatomical classification of sympathetic and parasympathetic [6, 8–10]. Dale and Loewi were awarded the 1936 Nobel Prize in Physiology or Medicine ‘for their discoveries relating to chemical transmission of nerve impulses’ [3]. While physiologists consider Dale a prominent physiologist, several of his works also touch upon anatomy, pharmacology, and therapeutics [4, 10].

In 1914, Dale was appointed director of the Department of Biochemistry and Pharmacology at the National Institute for Medical Research in London and was elected Fellow of the Royal Society [3]. In 1928, he became the Institute director, a role he kept until 1942; he was knighted 6 years later. He was inducted into the National Academy of Sciences of the USA in 1940, appointed Fullerian Professor of Chemistry at the Royal Institution and director of the Davy-Faraday Laboratory in 1942. Further, he received the Knight Grand Cross of the Order of the British Empire in 1943 and the Order of Merit in 1944.

Sir Henry Dale held an impressive series of high-profile leadership roles. In 1945, he concluded a 5-year service as president of the Royal Society after being one of the honourary secretaries with notable editorial commitments [8]. During the Second World War, he sat on several advisory panels to the Cabinet. He became president of the British Association in 1947, he was president of the Royal Society of Medicine for 1948–1950 (first non-practising president ever) and of the British Council for 1950–1955. He was a trustee of the Wellcome Trust since 1936 (as well as chairman of the Board for 1938–1960), ‘guiding with wisdom and foresight the spending of hundreds of thousands of pounds in encouraging medical research’, and promoted its Historical Medical Museum and Library [1]. His name is attached to the Sir Henry Dale Fellowship for young scientists (now discontinued) and the Dale Medal awarded by the Society for Endocrinology.

His many honours include the Royal and Copley Medals of the Royal Society, the Gold Albert Medal of the Royal Society of Arts, the Baly Medal of the Royal College of Physicians, the Medal of Freedom (Silver Palm), and more than 20 honourary degrees. Sir Henry Dale died in Cambridge on 23 July 1968.
